# Robust Design of SAW Gas Sensors by Taguchi Dynamic Method

**DOI:** 10.3390/s90301394

**Published:** 2009-02-27

**Authors:** Hsun-Heng Tsai, Der Ho Wu, Ting-Lung Chiang, Hsin Hua Chen

**Affiliations:** 1 Department of Biosystems Engineering, National Pingtung University of Science and Technology. Hseuh Fu Road, Neipu Hsiang, Pingtung, Taiwan, R.O.C. 91207; 2 Department of Mechanical Engineering, National Pingtung University of Science and Technology. Hseuh Fu Road, Neipu Hsiang, Pingtung, Taiwan, R.O.C. 91207; E-Mails: derhowu@mail.npust.edu.tw; tchiang@mail.npust.edu.tw

**Keywords:** Surface Acoustic Wave (SAW), Finite Element Method (FEM), Dynamic Taguchi Method, Signal-to-Noise ratio

## Abstract

This paper adopts Taguchi’s signal-to-noise ratio analysis to optimize the dynamic characteristics of a SAW gas sensor system whose output response is linearly related to the input signal. The goal of the present dynamic characteristics study is to increase the sensitivity of the measurement system while simultaneously reducing its variability. A time- and cost-efficient finite element analysis method is utilized to investigate the effects of the deposited mass upon the resonant frequency output of the SAW biosensor. The results show that the proposed methodology not only reduces the design cost but also promotes the performance of the sensors.

## 1. Introduction

Owing to its many advantages of high sensitivity, simplicity, low cost and ability to perform rapid measurements, the piezoelectric quartz crystal resonator has been widely used as a mass sensitive detector in electrochemical experiments recently. The application of an alternating electrical field perpendicular to the surface of the piezoelectric quartz crystal (PQC) induces a mechanical vibration of the piezoelectric surface, whose frequency is changed by loading effects generated when a small mass is deposited on the resonator surface [1–3]. Previous studies have shown that surface acoustic wave (SAW) sensors provide a superior resolution than quartz crystal microbalance (QCM) devices due to their higher operating frequencies of 100 to 200 MHz. SAW delay-line sensors have attracted particular attention since they are characterized by high sensitivity, up to 10^−9^ ∼ 10^−15^ g, a rapid response, low cost, small physical size, and a straightforward fabrication process [4,5]. The output response of the SAW device varies as a linear function with the input signal, which corresponds to the deposited mass, and some researchers have successfully optimized their mass sensitivity by considering the calibration curve of device response versus mass concentration [6–8].

SAW sensors consist of a thin ST-cut quartz disk sandwiched between metal electrodes and then coated with sensitive membranes. Traditionally, the design and development of these devices has relied heavily upon an experimental approach. However, the effects of operative error, or of faulty apparatus, are virtually impossible to eliminate in such a case. Consequently, discrepancies frequently exist between the design specification and the experimental results. Modern computer-aided design finite element method (FEM) techniques provide powerful simulation tools for the task of designing piezoelectric systems [9–10]. These techniques facilitate coupled-field finite element analysis and are capable of generating excellent results. The use of tools of this type provides an engineer with the ability to develop highly accurate predictions of a system’s likely performance, without the need to fabricate a physical prototype [11–12].

The Taguchi robust design method enables the main effects of certain designated design parameters to be evaluated. This method ensures the reproducibility of the experimental results and enables the optimum combination of design parameters (i.e. the control factors) to be determined from a minimum number of experiments. Taguchi parameter design can be divided into static and dynamic cases, in which the former case has no signal factor, while the latter has signal factors for the output optimization goals. Generally speaking, the accuracy of a measurement system is influenced by dynamic characteristics such as time-varying input signals or by the presence of noise [13]. Recently, Wu [14,15] successfully integrated a static model and a commercial FEM package to simulate the QCM and SAW systems. However, this study ignored the sensitivity considerations relating to mass effects (i.e. the signal factors) and noise factors. Hence, the robustness of the measuring system was not assured. Dynamic methods enable the measuring system to be optimized with an enhanced sensitivity over a range of output values, and therefore yield a more robust solution. This study integrates computer-aided simulation experiments with the Taguchi dynamic method to generate a robust SAW gas sensor design. The main objective of the proposed methodology is to reduce design and development costs and to enhance the robustness of the biosensor measuring performance.

## 2. Basic Piezoelectric Theory

### 2.1. SAW Mass Effect

**Figure 1.**
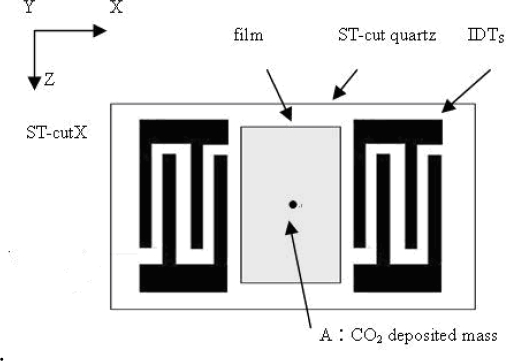
Schematic of SAW sensor model.

**Figure 2.**
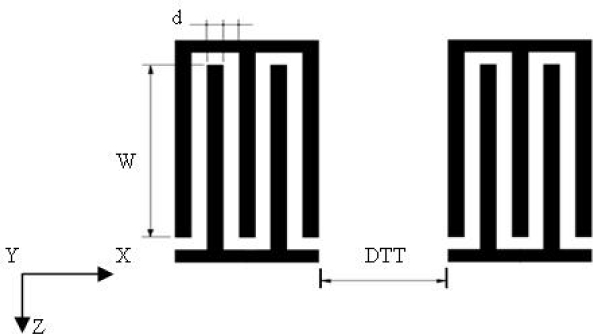
IDT_S_ structure.

Surface acoustic wave sensors are highly sensitive to mass changes on their surfaces. Even the deposition of a small mass on the surface of ST-cut quartz crystal in air causes a reduction of its original resonant frequency as shown in Figure 1. This frequency shift is proportional to the deposited mass per unit area of the sensing film. Ignoring oscillation circuit stability considerations, the higher the resonant frequency of the device leads to the smaller the mass variation is capable of detecting. SAW delay-line type devices are used in many mass-sensing applications. The Rayleigh wave type can be excited using an interdigital transducer (IDT). In this technique, the spatially periodic field of the IDT produces a periodic mechanical strain pattern [16] which causes acoustic waves to propagate away from either side of the IDT in a direction essentially perpendicular to the interdigital alignment of the transducer electrodes. As shown in Figure 2, the delay-line device consists of two IDTs with a constant electrode overlap, w, and a separation distance, L, implemented on an ST-cut quartz piezoelectric substrate. The operating resonant frequency of a SAW sensor is strongly related to the period of the IDT transducer. The IDT operates most efficiently when the acoustic wavelength of the SAW matches the transducer period.

The resonant frequency shift of a SAW sensor is directly proportional to the deposited mass per unit area, and hence provides an indication of the mass sensitivity of the device. In general, the sensitivity, *S*, of a gas sensing device is given by *S = dR/dn*, where *R* is the device response and *n* is the gas concentration. A device that develops a higher value of R or a greater frequency shift than other devices for the same deposited mass possesses a superior sensitivity. The response *R* for an uncoated substrate is defined as [16]:

(1) $$R=\frac{\Delta v}{v}=\frac{\Delta f}{f_{0}}=(k_{1}+k_{2})\;f_{0}\frac{\Delta m}{A_{s}}$$

where *v* is the phase velocity of the acoustic wave, k_1_ =−9.33×10^−8^ m^2^s/kg, k_2_ = −4.16×10^−8^ m^2^s/kg are the mass sensitivity constant, *f*_0_ is resonant frequency, and *A_s_* is the area of the coated-film.

### 2.2. Taguchi Dynamic Method

**Figure 3.**
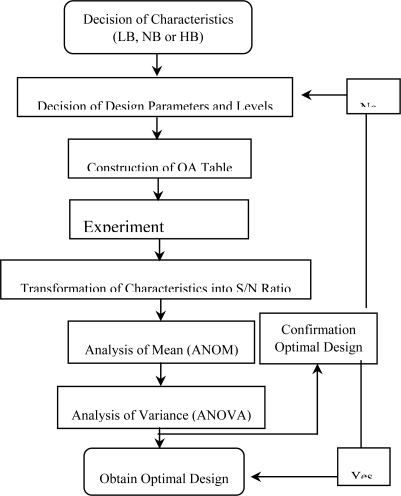
Procedure of Taguchi analysis.

Studies have shown that a robust measurement system has the following capabilities: 1) it minimizes variability as the input signal changes, 2) it provides consistent measurements for the same input, 3) it continues to give an accurate reading as the input values changes, 4) it adjusts the sensitivity of the design in transforming the input signal into an output, and 5) it is robust to noise [17,18]. Figure 3 presents a simplified representation of the dynamic measurement system. The input (signal) is the item which is to be measured, while the output is the value observed from the measurement system. The introduction of noise effects into the system causes the observed value to deviate slightly from the true value. Therefore, when designing the measurement system, it is necessary to develop a robust design with dynamic characteristics by utilizing Taguchi’s signal-to-noise (S/N) ratio to ensure the optimum design conditions. Generally, a dynamic study involves a two-step optimization procedure, in which initially the variation around a linear function is minimized, and secondly the sensitivity of the linear function is adjusted to a target value. The aim of the robust design is to adjust the control factor settings such that the system becomes less sensitive to variations in the noise effects. In order to achieve the desired output range or to meet the target sensitivity, it may be necessary to adjust the sensitivity of the response to the input signal value. An appropriate setting of the control factors enables the slope of the linear function between the output response and the signal factor to be adjusted as required. The linear nature of the relationship between the output response and the input signal is readily visualized and simplifies the task of making the necessary adjustments to the input signal so as to produce the desired output. In considering dynamic relationships, the zero-point proportional equation provides a useful tool to adjust the output by changing the input signal factor. This equation expresses a simple linear relationship between the response, *Y*, the signal factor, *M*, and the error, ε [19], i.e.

(2) $$Y_{\mathrm{ijk}}=\beta_{i}M_{j}+\varepsilon_{\mathrm{ijk}}$$

where the control factor is *i* = 1, 2, *I*, the signal factor is *j* = 1, 2, *J,* and the noise factor *k* = 1, 2, *r_0_*.

This equation describes a straight line of slope *β* passing through the zero point. An ideal piezoelectric biosensor should have a purely linear response and should have the ability to adjust its output, *Y* (i.e. the frequency shift), by changing the signal factor, *M* (i.e. the deposited mass), with a nonzero slope.

The dynamic S/N ratio is closely related to the static case and can be expressed conceptually in mathematical form as:

(3) $$S/N_{i}=10\;{\text{log}}_{10}\;(\frac{\beta_{i}^{2}}{{\mathrm{MSE}}_{i}})$$

where *β,* is the slope as determined by the least squares method (LSM). The LSM minimizes the sum of the squares of the data around a best fit and is expressed as follows:

(4) $$\beta_{i}=\frac{\sum_{j=1}^{J}\sum_{k=1}^{\mathrm{ro}}y_{\mathrm{ijk}}M_{j}}{r_{o}\sum_{j=1}^{J}M_{j}^{2}}$$

where *y_ij_* is the *jth* characteristic result of the *ith* experiment, *M_j_* is the j*th* level input signal, *r_o_* is the experimental trial number of the outer orthogonal array, and j is the level setting of the input signal.

In Equation (3), *MSE_i_* is the mean square error for the ith factor and is given by:

(5) $${\mathrm{MSE}}_{i}=\frac{1}{r_{o}J-1}\sum_{j=1}^{J}\sum_{k=1}^{\mathrm{ro}}{\left(y_{\mathrm{ijk}}-\beta_{i}M_{j}\right)}^{2}$$

## 3. Dynamic Robust Design of SAW Gas Sensor

### 3.1. Dynamic Robust Design

Dynamic robust design is an engineering methodology which renders a product or a process insensitive to the effects of variability. This methodology is applied during the research and development stage to ensure that high-quality products can be produced quickly and at low cost. The Taguchi method is an established robust design technique which has been successfully applied to the development of many products. The fundamental objective of robust design is to optimize the product and process designs such that they become insensitive to variations in the uncontrollable noise sources without actually eliminating these sources. The Taguchi method incorporates two principal tools, namely a S/N ratio to measure the quality of the design and an orthogonal array (OA), which permits the simultaneous consideration of many design parameters. In the SAW sensor, the frequency shift is related to the deposited mass via a linear equation (1) in which the deposited mass is regarded as the input signal (*M*) and the resonance frequency shift is considered to be the output response (*Y*). Meanwhile, the parameter *K* can be treated as the sensitivity of the linear equation, i.e. its slope, *β*. Increasing the value of *β* enhances the sensor sensitivity, while enlarging the S/N ratio reduces the variance induced by external noise.

In the SAW design, the frequency shift value is treated as the characteristic value and is ideally as large as possible in order to enhance the detection capabilities of the device. Therefore, the present robust design case is defined as a dynamic larger-the-better problem and the main objective of the design activity is to maximize the S/N ratio defined in Equation (3). Figure 3 presents the robust design procedure adopted in the present study. Meanwhile, Table 1 presents the specified SAW control factors and their respective level settings. This study adopts an L_18_(2^1^×3^7^) orthogonal array as Table 2, which is known to be less affected by interactions between the various design parameters. In a parameter design experiment, the control factors are assigned to an inner array, while the signal factor and noise interference factors are configured in an outer array. In the present study, the outer array consists of a signal factor with three levels, i.e. deposited mass values of: *M_1_*: 3.5728×10^−9^ g, *M_2_*: 3.5728×10^−8^ g, and *M_3_*: 3.5728×10^−7^ g, crossed with a three-level noise factor, i.e. *N_1_* = 41.75^0^, *N_2_* =42.75^0^, and *N_3_* = 43.75^0^, where N is the cut angle of the quartz. It is noted that these noise factors represent the dimensional errors in quartz crystal cutting angle introduced during manufacturing.

### 3.2. Computer Simulation

In accordance with the design parameter combinations in the OA table, eighteen different SAW finite element models were constructed using the model presented previously by Wu [14]. An ANYSY electro-mechanical couple-field solid 98 3D-element was utilized to account for the interaction between the structural and electric fields of the SAW device in the computer simulation, i.e. to model the voltage generated by the displacement and vibration of the piezoelectric material when subjected to an applied voltage. Variational principles were used to develop the finite element equations incorporating the piezoelectric effects and the electromechanical constitutive equations [20] in order to describe the linear material behavior, i.e.

(6) $$\left\{\begin{array}{c} \left\{T\right\}\\ \left\{D\right\} \end{array}\right\}=\left[\begin{array}{cc} \left[C\right] & \left[e\right]\\ {\left[e\right]}^{T} & -\left[\varepsilon \right] \end{array}\right]\left\{\begin{array}{c} \left\{S\right\}\\ -\left\{E\right\} \end{array}\right\}$$

where {*T*} is the stress vector, {*D*} is the electric displacement, {*S*} is the strain vector, {*E*} is the electric field vector, [*e*] is the piezoelectric coefficient, and [ε] is the dielectric matrix.

**Figure 4.**
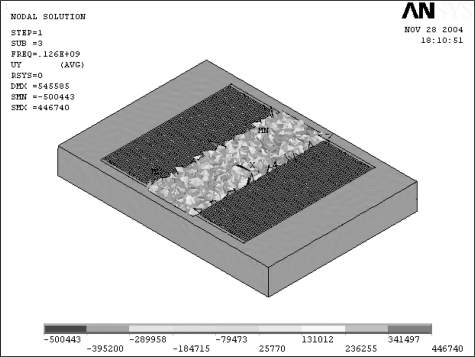
SAW sensor 3D geometry model.

**Figure 5.**
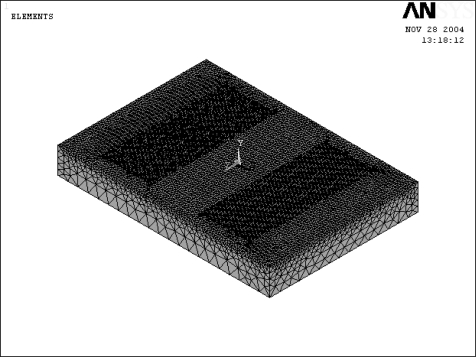
SAW sensor 3D FE model.

**Figure 6.**
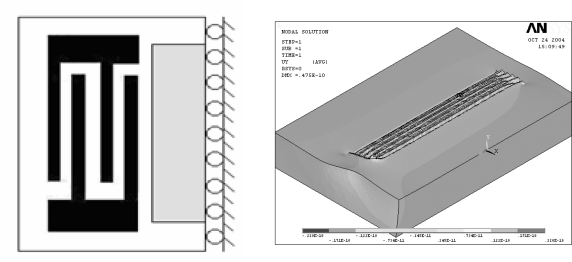
(a) Half FEM model (b)FE simulation of Rayleigh wave propagation of SAW.

Figures 4 and 5 present the geometrical model and the corresponding finite element mode of the SAW l, respectively. Meanwhile, Figure 6 shows the half-model TSM mode shape of the analyzed SAW is used in this study. Finite element simulations were performed for each of the 18 experimental trials of the OA table. The adopted anisotropic material properties of ST-cut quartz are provided in the Appendix for reference purposes. Having completed the finite element simulations, Equations (3)–(5) were applied to obtain the corresponding S/N ratio and βvalues for each trial run. The corresponding results are presented in Table 3.

## 4. Two-Step Taguchi’s Dynamic Analysis

### 4.1. Optimization of Control Factors

**Figure 7.**
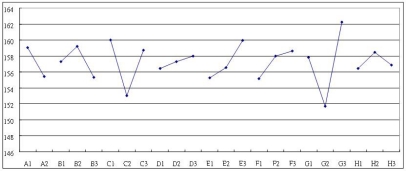
The S/N cause effects graph of control factors.

**Figure 8.**
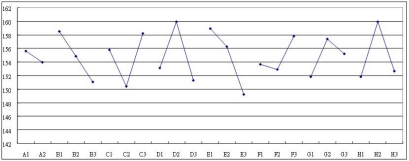
The gain cause effects graph of control factors.

In the two-step optimization of the current larger-the-better dynamic problem, the quality is first optimized by identifying the control factors which significantly influence the S/N ratio and the sensitivity, and then appropriate control factor level settings are established to reduce the variability and to increase the sensitivity of the measurement system. Two statistical analysis methods, namely, the Analysis of Mean (ANOM) and the Analysis of Variance (ANOVA) are utilized to establish the optimum design conditions. In order to obtain the optimum combination of design parameters, the control factor effects are analyzed using ANOM to identify the factors which are primarily responsible for inducing variation in the S/N ratio and in the sensitivity. Figures 7 and 8 indicate the magnitudes of the average response effects of the various control factors for the S/N ratio and for the sensitivity, respectively.

The results of these figures enable the optimum level of each control factor to be identified. The ANOVA approach is a mathematical technique commonly known as the sum of squares. Using this method, the relative contribution of each control factor can be estimated quantitatively and the overall measured response can be expressed as a percentage. In this study, ANOVA is used to identify the control factors which significantly reduce the variability and which bring the sensitivity toward its target value. Table 4 provides an ANOVA analysis of the Taguchi S/N ratio. Table 5 indicates that Factors B,C and E have a significant effect upon the sensitivity and reduce variability. Factors A and G are influential in reducing variability. Meanwhile, Table 5 indicates that Factors D and H have a significant effect upon the sensitivity. The results confirm that the thicknesses of the senor membranes play an important role in determining the sensitivity.

Having identified the factors which have a significant influence on the S/N ratio and sensitivity, appropriate settings of each design parameter must be chosen in order to reduce the variability and increase the sensitivity. However, if the design objective is to maximize the device sensitivity β and reduce variability, then from Figure 7 and Figure 8, the appropriate factor level settings are A_1_B_2_C_1_D_2_E_1_F_3_G_3_H_2_. It can be seen that there is a contradiction between these two sets of results, and hence some degree of compromise is necessary. Of the five control factors, factor F can be considered as an adjustable factor whose setting is dependent on economic or manufacturing considerations.

### 4.2. Prediction and Verification

**Figure 9.**
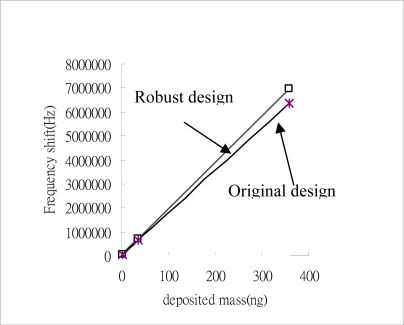
Comparisons of original and robust design for sensitivity β analysis.

The aim of this step is to verify that the optimum control factor treatment combination established in the above analysis is correct. It has been shown that the optimum factor level settings depend on whether it is the S/N ratio or the sensitivity β which is to be optimized. Two separate tests are conducted for verification purposes. In the first test, an additive model is used for prediction purposes, while in the second, a simulation experiment is performed. The optimum factor level settings for the original design are found to be A_2_B_2_C_2_D_2_E_2_F_2_G_2_H_2_, which compare to A_1_B_2_C_1_D_2_E_1_F_3_G_3_H_2_ for the robust dynamic design. As shown in Table 6, a discrepancy exists between the predicted and verified results for the S/N ratio and the sensitivity for both the original and the robust design. The results demonstrate that the Taguchi dynamic characteristic design process successfully increases both the S/N ratio and the sensitivity of the SAW. Hence, the biosensor is capable of detecting smaller mass changes within the same limit of the frequency counter without a loss in reliability or precision. This result is presented graphically in Figure 9.

## 5. Conclusions

The purpose of this paper has been to establish a dynamic measurement system design for a piezoelectric SAW biosensor. The following brief conclusions can be drawn:

1. This study represents the first time that the Taguchi dynamic design method has been integrated with computer simulation in the study of SAW devices. The results indicate that the adopted methodology enables the device sensitivity to be increased while reducing its variability.
2. FEM simulation is convenient, rapid, accurate, inexpensive, and straightforward to implement and learn. This technique provides a new and effective coupled field piezoelectric design tool.
3. The present robust dynamic design has confirmed that the control factors such as the thickness of sensor membrane, DDT, electrode thickness, No. of electrode finger pairs and the electrodes are essential design parameters in that they significantly influence the precision and sensitivity of the SAW biosensor. It is noted that for reasons of simplicity, this study has considered only a single noise factor. It is recommended that future studies implement more noise factors in order to reflect a more realistic working environment.

## Figures and Tables

**Table 1.** Control factor and level.

**Control factor**	**Level 1**	**Level 2**	**Level 3**

A Types of ST quartz	ST-cutX Quartz	ST-cutY Quartz	
B No. of electrode finger pairs	20	40	60
C Delay distance (×10^−3^ m)	95λ = 2.28	100λ = 2.4	105*λ* = 2.52
D Electrode thickness *h_e_* (Å)	1800	1900	2000
E Electrode overlay W(×10^−3^ m)	5.76	6	6.24
F Types of sensor membrane	rubbery	glassy-rubbery	glassy
G Sensor membrane thickness *h_f_* (μm)	0.21	0.22	0.23
H dimensions of matrix(×10^−3^ m)	9.8×6.8×1	10×7×1	10.2×7.2×1

**Table 2.** L_18_(2^1^×3^7^) Orthogonal array.

**No. Exp.**	**Control Factor**							
	**A**	**B**	**C**	**D**	**E**	**F**	**G**	**H**
**1**	1	1	1	1	1	1	1	1
**2**	1	1	2	2	2	2	2	2
**3**	1	1	3	3	3	3	3	3
**4**	1	2	1	1	2	2	3	3
**5**	1	2	2	2	3	3	1	3
**6**	1	2	3	3	1	1	2	2
**7**	1	3	1	2	1	3	2	3
**8**	1	3	2	3	2	1	3	1
**9**	1	3	3	1	3	2	1	2
**10**	2	1	1	3	3	2	2	1
**11**	2	1	2	1	1	3	3	2
**12**	2	1	3	2	2	1	1	3
**13**	2	2	1	2	3	1	3	2
**14**	2	2	2	3	1	2	1	3
**15**	2	2	3	1	2	3	2	1
**16**	2	3	1	3	2	3	1	2
**17**	2	3	2	1	3	1	2	3
**18**	2	3	3	2	1	2	3	1

**Table 3.** Simulation data of dynamic analysis.

**No.**	***M*_1_**			***M*_2_**			***M*_3_**		
	**N_1_**	N_2_^*^	N_3_^*^	**N_1_**	**N_2_**	N^*^	N_1_^*^	N_2_^*^	N_3_^*^
**1**	5.9544	5.6137	4.9526	59.5442	56.1371	49.5256	595.4416	561.3709	495.2558
**2**	7.1123	5.7219	4.9320	71.1227	57.2190	49.3202	711.2268	572.1900	493.2021
**3**	5.5450	5.5383	5.3513	55.4500	55.3828	53.5127	554.4994	553.8276	535.1274
**4**	5.4662	5.3247	5.2557	54.6623	53.2474	52.5567	546.6235	532.4737	525.5666
**5**	5.4200	5.1293	4.9677	54.1996	51.2925	49.6774	541.9964	512.9252	496.7736
**6**	6.3338	5.8331	5.3219	63.3379	58.3312	53.2185	633.3791	583.3116	532.1852
**7**	6.8546	6.0335	5.3645	68.5457	60.3350	53.6445	685.4569	603.3505	536.4451
**8**	5.7614	5.2287	4.7018	57.6145	52.2869	47.0182	576.1448	522.8694	470.1817
**9**	5.7140	5.4216	5.2758	57.1402	54.2158	52.7585	571.4015	542.1579	527.5847
**10**	5.7761	5.2158	4.9665	57.7609	52.1579	49.6647	577.6092	521.5794	496.6465
**11**	6.4332	5.8204	5.5289	64.3322	58.2037	55.2894	643.3219	582.0373	552.8947
**12**	6.3500	5.8675	5.2588	63.5002	58.6746	52.5882	635.0022	586.7465	525.8818
**13**	5.9821	5.7190	5.6693	59.8209	57.1903	56.6933	598.2086	571.9030	566.9328
**14**	5.9668	5.2947	4.4197	59.6676	52.9465	44.1973	596.6765	529.4654	441.9732
**15**	6.8332	5.5468	5.0760	68.3321	55.4676	50.7604	683.3214	554.6763	507.6042
**16**	5.6545	5.3507	5.0336	56.5454	53.5073	50.3358	565.4542	535.0729	503.3581
**17**	6.1350	5.7287	5.4108	61.3502	57.2874	54.1076	613.5021	572.8741	541.0759
**18**	5.8872	5.4612	5.1292	58.8720	54.6125	51.2920	588.7199	546.1248	512.9201

**Table 4.** S/N Dynamic results.

**No.**	***MSE***	***β*(10^11^)**	***S / N***	**No.**	***MSE***	***β*(10^11^)**	***S / N***

**1**	255982.28^2^	154.13	155.59	**10**	208368.03^2^	148.89	157.08
**2**	554690.05^2^	165.75	149.51	**11**	231941.75^2^	165.91	157.09
**3**	55255.11^2^	153.33	168.87	**12**	274784.63^2^	163.05	155.47
**4**	53940.54^2^	149.71	168.87	**13**	84458.61^2^	162.06	165.66
**5**	115161.77^2^	144.77	161.99	**14**	389818.28^2^	146.30	151.49
**6**	254263.75^2^	163.17	156.15	**15**	457088.89^2^	162.86	151.04
**7**	375054.86^2^	170.29	153.14	**16**	156034.23^2^	149.64	159.64
**8**	266243.53^2^	146.40	154.81	**17**	901749.61^2^	133.18	143.39
**9**	112131.85^2^	153.11	162.71	**18**	190940.61^2^	153.73	158.12

				**Avg.**	274328.24^2^	154.79	157.25

**Table 5.** The results of ANOVA.

**S/N ratio**				**Gain**			

**Factor**	***SS***	***DOF***	***Var***	**Factor**	***SS***	***DOF***	***Var***

**A**	59.2562	1	59.2562	**A**	12.5668	1	12.5668
**B**	45.6002	2	22.8001	**B**	166.5850	2	83.2925
**C**	164.4157	2	82.2078	**C**	192.4940	2	96.2470
**D**	7.3055	2	3.6528	**D**	248.9454	2	124.4727
**E**	70.2703	2	35.1351	**E**	300.8786	2	150.4393
**F**	40.1874	2	20.0937	**F**	83.0479	2	41.5239
**G**	334.6497	2	167.3248	**G**	92.9377	2	46.4689
**H**	13.5930	2	6.7965	**H**	240.5441	2	120.2721
**Others**	17.9588	2	8.9794	**Others**	205.9727	2	102.9864

**Total**	753.2366	17		**Total**	1543.9724	17	

**Table 6.** The comparisons of S/N ratio between original design and Taguchi design.

	**original design**	**Taguchi design**	**Improvement**
***β* (10^11^)**	163.81	175.26	11.45
***S/N* (db)**	149.89	170.08	20.20

## Appendices

Stiffness matrix of Quartz

$$\left[C_{\mathrm{pq}}^{E}\right]=\left[\begin{array}{cccccc} 86.74 & 6.99 & 11.91 & -17.91 & 0 & 0\\ 6.99 & 86.74 & 11.91 & 17.91 & 0 & 0\\ 11.91 & 11.91 & 107.20 & 0 & 0 & 0\\ -17.91 & 17.91 & 0 & 57.94 & 0 & 0\\ 0 & 0 & 0 & 0 & 57.94 & -17.91\\ 0 & 0 & 0 & 0 & -17.91 & 73.43 \end{array}\right]\times 10^{9}\frac{N}{m^{2}}$$

Piezoelectric strain matrix of Quartz

$$\left[e_{\mathrm{ij}}\right]=\left[\begin{array}{cccccc} 0.171 & -0.171 & 0 & 0.0436 & 0 & 0\\ 0 & 0 & 0 & 0 & -0.0436 & -0.171\\ 0 & 0 & 0 & 0 & 0 & 0 \end{array}\right]\frac{C}{m^{2}}$$

Permittivity matrix of Quartz

$$\left[\varepsilon_{\mathrm{iJ}}^{S}\right]=\left[\begin{array}{ccc} 3.984 & 0 & 0\\ 0 & 3.984 & 0\\ 0 & 0 & 4.073 \end{array}\right]\times 10^{-11}\frac{F}{m}$$

Piezoelectric stress matrix of ST-cutX

$$\left[e_{\mathrm{ij}}\right]=\left[\begin{array}{cccccc} 0.1710 & -0.0487 & -0.1223 & 0.0887 & 0 & 0\\ 0 & 0 & 0 & 0 & 0.0617 & -0.1139\\ 0 & 0 & 0 & 0 & -0.0571 & 0.1053 \end{array}\right]\frac{C}{m^{2}}$$

Permittivity matrix of ST-cutX

$$\left[\varepsilon_{\mathrm{iJ}}^{S}\right]=\left[\begin{array}{ccc} 3.984 & 0 & 0\\ 0 & 4.025 & 0.044\\ 0 & 0.044 & 4.032 \end{array}\right]\times 10^{-11}\frac{F}{m}$$
